# Increasing belief but issue fatigue: Changes in Australian Household Climate Change Segments between 2011 and 2016

**DOI:** 10.1371/journal.pone.0197988

**Published:** 2018-06-18

**Authors:** Mark Morrison, Kevin Parton, Donald W. Hine

**Affiliations:** 1 Faculty of Business, Justice and Behavioural Sciences, Charles Sturt University, Bathurst, Australia; 2 School of Behavioural, Cognitive and Social Sciences, University of New England, Armidale, Australia; George Mason University, UNITED STATES

## Abstract

Using national Australian samples collected in 2011 (n = 1927) and 2016 (n = 2503), we identified six Australian household segments which we labelled *Alarmed*, *Concerned*, *Cautious*, *Disengaged*, *Doubtful* and *Dismissive*. Between the two periods, we found the proportion of households in the *Alarmed* and *Concerned* segments was stable; however there was a decrease (28% to 20%) in the proportion of households in the *Doubtful* and *Dismissive* segments and an increase (27% to 33%) in the *Cautious* and *Disengaged* segments. We found that a greater proportion of households have personally experienced climate change, and were more likely to believe in human causation and believe that there is a scientific consensus about the issue. However, there was evidence of issue fatigue. Households were less likely to report that they had thought about climate change or talked about it with their friends in 2016 relative to 2011. They were also less likely to pursue certain climate friendly behaviours or reward or punish companies for their climate behaviours. These findings suggest a need to motivate households to maintain efforts to mitigate climate change, particularly the *Cautious* and *Disengaged* households that are more amenable to changing their views about this issue.

## Introduction

Engaging the public about climate change has proven to be a substantial ongoing challenge for scientists and policy makers. An important first step to effective engagement involves understanding the number and nature of the audiences one needs to target–a process known as audience segmentation. Segmentation involves specifying a population of interest and identifying homogenous subgroups that share similar demographic and/or psychographic profiles. Once a population has been segmented, climate change communicators can target their messages based on the distinctive characteristics of each subgroup. For example, to stimulate engagement and behaviour change, messages targeting audiences that are sceptical about climate change may require different content, frames, and delivery channels than messages aimed at audiences who are already alarmed about climate change and its impacts [1, 2]. The primary aim of the present study is to compare the climate change audience segments derived using Australian national data sets collected in 2011 and 2016, and identify implications from any changes that are observed.

According to Hine et al. [1], segmentation can assist climate change communicators to make four main strategic decisions:

1. *Who should be targeted*? Audience segmentation research reveals the number of distinct audiences present in a population, the characteristics of each audience, and their relative sizes. This information is essential for making strategic resource allocation decisions. For example, to optimise resource allocation and cost-effectiveness, an organisation may decide to bypass small, entrenched sceptical segments, and focus on larger uncertain or unconvinced segments who may be easier to engage [3].
2. *How to optimise messages and intervention programs for each audience selected for targeting*? Audience segments each embody a unique combination of demographic, psychological and behavioural attributes, which can be informative in crafting communications and other interventions designed to address the knowledge needs and behaviour-change barriers facing each segment. For example, some segments may be sceptical about the climate change science, whereas others may be motivated to act, but lack specific information about what they can do.
3. *How to ensure the messages and interventions reach selected audiences*? Audience segments sometimes have unique preferences about where they obtain information about climate change. Some rely heavily on Facebook, others watch cable news, and others prefer reading traditional print media. If climate change communicators use the wrong channels, key messages may fail to reach their intended recipients, and may elicit backlash effects if, for example, a climate message framed for an alarmed audience is presented to a highly sceptical or dismissive one.
4. *How to select messengers for each audience segment*? Not all climate change messengers will be perceived as equally credible and trustworthy by all audience segments. Divisive figures like former Australian Prime Ministers Tony Abbott and Julia Gillard are highly respected by some, and reviled by others. Audience segmentation can aid climate change communicators in selecting messengers with the relevant expertise, values and personal experiences needed to build and maintain trust with their particular audiences.

## Identifying audience segments relevant to climate change

Climate change researchers and communication practitioners are becoming increasingly interested in applying social marketing principles, including audience segmentation, to better understand their audiences. Much of this climate change segmentation research has focused on identifying groups that share similar beliefs, attitudes, and behaviours related to climate change within large national samples. The “Climate Change in the American Mind” collaboration between researchers at the Yale Program on Climate Change Communication and the George Mason Center for Climate Change Communication, is the most influential and well-established of such programs. Data for its initial segmentation study were collected from a large nationally representative sample of US residents in 2008 [4]. Segmentation based on 36 variables assessing climate change beliefs, issue involvement, policy preference and behaviours revealed six distinctive segments: *Alarmed*, *Concerned*, *Cautious*, *Disengaged*, *Doubtful*, and *Dismissive*. Collectively labeled the *Six Americas*, the segments reflect quantitative shifts from generally high to generally low levels of concern, and degree of certainty that global warming is occurring, with engagement having a curvilinear relationship such that it is highest among those who are in segments at either end of this continuum–that is *Alarmed* and *Concerned*, or *Doubtful* and *Dismissive*–but lowest among the *Cautious* and *Disengaged* (see also [5]).

Follow-up studies by the Yale/George Mason group have monitored how the proportion of respondents in each segment has changed across time [4–14]. Segment proportions fluctuated somewhat over the 8-year period from 2008 to 2016, but there was no strong temporal trend toward increasing denial or acceptance of climate change. By 2016, membership of the *Alarmed* segment had increased after a slump and returned to 2008 levels.

Large national samples from Australia [15, 16, 3], India [17], and Germany [18] have been segmented using variations of the Yale/George Mason group’s measures and methodology. In an Australian comparative study, Morrison et al. [16] found that the climate change attitudes and behaviours of Australians were less polarised than US residents, with fewer respondents classified as *Alarmed* or *Concerned* and more classified in the centrist *Cautious* and *Disengaged* groups.

Segmentation of a sample of the Indian population, using a subset of the Yale/George Mason group’s measures, identified six segments: *Informed*, *Experienced*, *Undecided*, *Unconcerned*, *Indifferent*, and *Disengaged*. Notably, the proportion of *Disengaged* respondents in India was considerably larger in rural settings (19%) than in urban settings (10%) [17]. Employing a representative sample of German residents, Metag et al. [18] factor-analysed responses to a set of questions that consisted primarily of the Yale/George Mason Group’s measures. Cluster analysis of the resulting seven factor scores identified five segments that were collectively labeled the *Five Germanys*: *Alarmed*, *Concerned Activists*, *Cautious*, *Disengaged*, and *Doubtful*. This finding revealed a relatively high degree of concern about climate change among German respondents. Of particular interest was the finding that the highly dismissive segment present in the US, Australian and Indian samples was absent in Germany.

Several studies have incorporated a broader range of psychological variables, some of which are not explicitly related to climate change, in their segmentation analyses. For example, in another Australian study, Hine et al. [19] used a broad set of profiling variables including environmental values, trust, emotional responses and spatial and temporal discounting, in addition to the more standard climate change belief variables used in other studies. They identified five segments (*Dismissive*, *Doubtful*, *Uncertain*, *Concerned and Alarmed*) and found significant differences across segments on a range of validation dimensions, including climate change mitigation behaviours and energy policy preferences. In their study, the *Uncertain* segment shared characteristics of the *Disengaged* and *Cautious* segments identified in other studies in the US and Australia, but it is difficult to make meaningful comparisons across the studies because this study used a different set of measure to define segments. In the UK, the Department for Environment, Food and Rural Affairs (DEFRA) segmented 3600 English residents based on their attitudes, beliefs and behaviours about environmental issues, including (but not restricted to) climate change. Seven segments were identified (*Positive Greens*, *Waste Watchers*, *Concerned Consumers*, *Sideline Supporters*, *Cautious Participants*, *Stalled Starters*, and *Honestly Disengaged)*, which varied in terms of unique motivations and barriers, and also in the degree to which they engaged in climate change mitigation behaviours [20].

In addition to the studies cited above, numerous other segment-identification studies have been conducted across the world using a broad range of methodologies and theoretical frameworks. Hine et al. [2] provide a review of these studies.

## Engaging climate change audience segments

Although most climate change segmentation studies have focused on identifying segments and how they change over time, researchers are becoming increasingly interested in determining how to best engage with the audience segments they identify. Morrison et al. [21] examined how to engage household segments in climate change policy. They found the perceived trustworthiness of celebrities, scientists and left-wing politicians decreased steadily across the segments from *Alarmed* to *Dismissive*. They argued that the *Cautious* segment was the most politically-salient voter segment because its members were relatively open to changing their climate change views and were generally supportive of both government and opposition policies.

Morrison’s research group [3] further investigated cautious respondents by identifying cautious sub-segments (*Stay-at-home Parents*, *Professionals*, and *Retirees*), and evaluating each sub-segment’s responsiveness to marketing stimuli. Overall, the word “farmer” and images of farmers evoked positive emotions and a desire to act, and only one call to action—“*Deteriorating atmosphere is a major issue in the world today*”—was received positively by all cautious respondents.

In another Australian study focusing on how message content influences climate change adaptation intentions, Hine et al. [22] found messages with strong negative emotive content or that provided specific adaptation advice increased adaptation intentions in all three of their viewer segments (*Dismissive*, *Uncertain*, and *Alarmed*). The study also found that including information about local impacts and not mentioning climate change was effective in increasing engagement in *Dismissive* audiences. Similarly, in another Australian study, Bain et al. [23] demonstrated that pro-environmental messages framed in terms of social welfare and economic development, as opposed to avoiding risks associated with climate change, were more likely to be accepted by *Climate Change Deniers*.

Using the DEFRA segmentation framework, Horton and Doran [24] found that shifts between segments could be facilitated by targeting individuals’ beliefs about fairness related to sustainable consumption and climate change. Following focus group sessions, numbers of individuals classified as *Positive Greens* and *Waste Watchers* increased and respondents classified as *Stalled Starters* and *Disengaged* decreased. Flora et al. [25] found that exposing high school students to an engaging 50-minute entertainment-education presentation on climate science increased their knowledge of climate science, positive engagement with climate change, and almost all assessed conservation behaviours. Maibach et al.’s [26] segmentation tool was used to group students into the Six Americas audience segments approximately 2.5 days before and after the edutainment. Thirty-eight percent of students moved into a more engaged segment after the edutainment, whereas only 13% moved to a less engaged segment. The largest shifts into more engaged segments were from the initially *Disengaged* and *Doubtful* groups.

## Current study

The current study extends the Australian climate change audience segmentation literature by exploring how segment membership has changed from 2011 to 2016 in comparable Australian samples using the Yale/George Mason group’s Six America’s classification system. Longitudinal segmentation enabled us to quantitatively assess the extent to which climate change perceptions, policy preferences and behaviour have shifted during the past five years, and also the uniformity of these shifts across various demographic and political subgroups (e.g., urban versus rural, Coalition versus Labor voters, etc.). This will provide practical, actionable information to help climate change communicators to more effectively craft and target their messages. Our focus on understanding community behaviours as well as attitudes also answers the call of Kahan and Carpenter [27] for more field-based studies, as well as the call of Levine and Kline [28] to recognize the importance of measuring not just attitudes, but behaviours as well, as these can diverge in unexpected ways.

## Method

### Sample

We surveyed 1,927 Australian respondents in August 2011 and 2,503 respondents in January and February 2016 selected from an online panel provided by the Online Research Unit. The Charles Sturt University Faculty of Business Ethics in Human Research Committee provided ethics approval for the survey work which involved an online questionnaire (protocol number 200/2015/20). Consent to participate was informed. The purpose of the survey was explained to respondents prior to starting the survey, and other details related to consent including that the survey was voluntary, and that they were free to not participate or to stop participating once they had started the survey. Respondents were told that they could stop participating at any time by closing their browser window. In addition, a statement at the end of the section providing information about the survey was included stating “If you wish to participate, click here”.

A two-stage probabilistic sampling procedure was used, with initial random sampling within the sample frame, and additional random sampling within specific sections of the sample frame to ensure representativeness of the population across gender and age. The final samples exclude those who rushed the survey (completed in less than 8 minutes, as this represented the minimum time to quickly read the survey and respond to the questions without simply randomly clicking responses).

The sociodemographics of the two samples are summarised in Table 1. The comparison reveals that the sociodemographic profiles of the samples are similar.

**Table 1:** Sociodemographic characteristics of the 2011 and 2016 samples.

	2011	2016
Age	47.4	46.9
Male	49.0%	47.7%
Degree qualified	29.5%	34.7%
Trade qualified	27.1%	23.4%
Income	$74,541	$76,528
Capital City Resident	57.6%	63.9%
Regional Town Resident	29.5%	24.4%
Employed full-time	35.6%	33.2%
Employed part-time or casual	15.7%	17.0%
Self employed	6.4%	5.5%
Unemployed	4.4%	4.7%
Student	3.5%	6.9%
Home duties / not in paid employment—not looking for work	11.3%	9.3%
Retired / pension recipient	23.1%	23.5%

### Questionnaire

Both our 2011 and 2016 surveys included the 36 items used in the survey developed by Maibach et al. [26] to identify climate change segments. These items have been used to identify household climate change segments in a range of countries, including the US, India, Australia and Germany [17, 16, 18]. While one goal of the overall research project was the segmentation of households, other sections of the questionnaire were designed to assess the willingness to pay of households for various policies, to examine the issue of improving the effectiveness of communications about climate change, and to compare the climate change beliefs and attitudes of different religious groups. Findings from our 2011 survey on segmentation where we compare Australia and the US are presented in Morrison et al. [16], while our results on communication are presented in Morrison et al. [21] and Sherley et al. [3], and our investigation of beliefs and attitudes towards climate change of different religious groups are presented in Morrison et al. [15].

### Data analysis

Both the 2011 and 2016 data were combined into a single data set for the analysis, so that segments were comparable across the two time periods. We follow the conventional approach of this type of survey work and ask respondents to represent their household. Thus while individuals complete the questionnaire, we analyse the results by household. Latent class analysis was run using Latent Gold 4.5 to identify segments [29]. We tested a range of segment solutions, and higher numbers of segments were generally better, based on a range of measures including AIC, BIC and log-likelihood. However, given the impracticality for policy makers of using a larger number of segments, the desire to compare the results of this study with our earlier findings, and because larger segmentation solutions generally do not produce substantively different segments to those found in smaller solutions, it was decided to use a six-segment solution. Note that this decision to use six rather than a larger number of segments parallels a similar decision made by Maibach et al. [26].

## Results

### Segments

The six segments identified were labelled *Alarmed*, *Concerned*, *Cautious*, *Disengaged*, *Doubtful* and *Dismissive*. As shown in Tables 1–4, they were profiled using 35 items based on climate change beliefs, issue involvement, preferred societal response, and energy use behaviours. The *Alarmed* segment followed by the *Concerned* segment have higher proportions who are certain that global warming is occurring, who accept human causation and consider that there is scientific consensus about global warming, whereas the *Doubtful* and particularly the *Dismissive* segments have the low proportions with these views. Members of the *Cautious* and *Disengaged* segments are those who could most easily change their mind about climate change. Further details are presented about each of these segments in the following.

**Table 2:** Climate change beliefs by segment and overall: 2011 and 2016.

	Year	Alarmed	Concerned	Cautious	Disengaged	Doubtful	Dismissive	Overall	Scale Range
Think climate change is happening^a^	2011 2016 *% Change*	8.67 8.75 *0*.*8%*	7.68 7.88 *2*.*7%*	6.51 6.69 *2*.*8%*	6.25 6.19 *-0*.*9%*	5.14 4.88 *-5*.*1%*	2.77 3.16 ***13*.*8%***	6.54 6.89 *5*.*4%*	9
Whether climate change is caused mostly by human activities^b^	2011 2016 *% Change*	88% 98% ***11*.*4%***	69% 88% ***27*.*5%***	45% 67% ***48*.*9%***	18% 41% ***127*.*8%***	5% 18% ***260*.*0%***	2% 2% *0%*	57% 65% ***13*.*3%***	—
Agreement between scientists^c^	2011 2016 *% Change*	81% 86% *6*.*2%*	54% 67% ***24*.*1%***	29% 41% ***41*.*4****%*	28% 37% *32*.1%^*^	13% 19% ***46*.*2%***	7% 8% ***14*.*3%***^***#***^	39% 51% ***30*.*8%***	—
How much will harm you personally^d^	2011 2016 *% Change*	3.26 3.29 *0*.*9%*	2.76 2.77 *0*.*4%*	2.18 2.22 *1*.*7%*	2.15 2.21 *2*.*8%*	1.71 1.57 *-8*.*1%*	1.06 1.07 *1*.*0%*	2.34 2.44 *4*.*3%*	4
How much will harm future generations^d^	2011 2016 *% Change*	3.96 3.97 *0*.*3%*	3.71 3.72 *0*.*1%*	3.23 3.23 *0*.*1%*	2.61 2.47 *-5*.*4%*	2.27 2.21 *-2*.*8%*	1.28 1.21 *-5*.*9%*	3.12 3.22 *3*.*1%*	4
How much will harm plant and animal species^d^	2011 2016 *% Change*	3.91 3.97 *1*.*4%*	3.62 3.70 *2*.*1%*	3.16 3.25 *2*.*9%*	2.63 2.48 *-5*.*5%*	2.35 2.23 *-4*.*8%*	1.27 1.24 *-2*.*3%*	3.07 3.21 *4*.*8%*	4
When climate change will harm people in Australia^e^	2011 2016 *% Change*	5.55 5.71 *2*.*8%*	4.87 5.08 *4*.*4%*	3.90 4.31 ***10*.*5%***	3.68 3.89 *5*.*5%*	2.65 2.74 *3*.*5%*	1.21 1.17 *-3*.*7%*	3.93 4.33 ***10*.*3%***	6
Humans capacity to reduce climate change^f^	2011 2016 *% Change*	3.87 3.71 *-4*.*0%*	3.79 3.66 *-3*.*5%*	3.59 3.45 *-3*.*7%*	3.16 2.93 *-7*.*3%*	2.54 2.52 *-0*.*7%*	1.63 1.55 *-5*.*1%*	3.27 3.26 *-0*.*3%*	5
Actions of single individuals wont make a difference^g^	2011 2016 *% Change*	3.36 3.31 *-1*.*4%*	2.96 2.93 *-1*.*1%*	2.57 2.59 *0*.*6%*	2.41 2.26 *-6*.*0%*	2.09 2.14 *2*.*6%*	1.57 1.77 ***12*.*6%***^***#***^	2.61 2.67 *2*.*5%*	4
New technologies will solve climate change without individuals having to make big changes in their lives^g^	2011 2016 *% Change*	1.84 1.87 *2*.*0%*	2.13 2.27 *6*.*2%*	2.33 2.43 *4*.*4%*	2.57 2.79 *8*.*4%*	2.49 2.52 *1*.*1%*	2.47 2.46 *-0*.*5%*	2.26 2.34 *3*.*4%*	4
Own actions will reduce personal contribution to climate change^h^	2011 2016 *% Change*	2.93 2.88 *-1*.*5%*	2.56 2.45 *-4*.*3%*	2.14 1.99 *-7*.*0%*	2.29 2.42 *5*.*9%*	1.59 1.61 *1*.*2%*	1.03 1.16 ***12*.*7%***	2.19 2.23 *1*.*9%*	4
If most people in Australia did these actions how much it would reduce climate change^h^	2011 2016 *% Change*	3.47 3.43 *-1*.*3%*	3.08 3.03 *-1*.*7%*	2.53 2.45 *-3*.*4%*	2.45 2.62 *7*.*1%*	1.70 1.76 *3*.*5%*	1.03 1.11 *7*.*8%*	2.53 2.64 *4*.*1%*	4
If most people in industrialised countries did these how much it would reduce climate change^h^	2011 2016 *% Change*	3.72 3.66 *-1*.*6%*	3.50 3.43 *-2*.*1%*	2.94 2.84 *-3*.*5%*	2.69 2.65 *-1*.*3%*	2.06 1.98 *-3*.*5%*	1.12 1.25 ***11*.*4%***	2.86 2.92 *2*.*1%*	4

Notes

^a^ 1 –extremely sure global warming is not happening, …, 9 –extremely sure global warming is happening

^b^ 1– caused mostly by human activities, 0 –caused mostly by natural changes in the environment, 0 –none of the above because climate change isn’t happening

^c^ 1 –most scientists think climate change is happening, 0 –most scientists think climate change is not happening, 0 –there is a lot of disagreement among scientists about whether or not climate change is happening

^d^ 1 –not at all, 4 –a great deal

^e^ 1—never, 2–100 years, 3–50 years, 4–25 years, 5–10 years, 6 –they are being harmed now

^f^ 1 –global warming isn’t happening, 2 –humans can’t reduce global warming, even it if is happening, 3- humans could reduce global warming, but people aren’t willing to change behaviour, 4—humans could reduce global warming, but it is unclear at this point whether we will do what’s needed, 5 –humans can reduce global warming, and we are going to do so successfully

^g^ 1 –strongly agree, …, 4 –strongly disagree

^h^ 1 –not at all, 2 –a little, 3—some, 4 –a lot

* significant difference at p<0.10

# nonsignificant difference, all other shaded cells have significant differences at p<0.05 or higher

**Table 3:** Climate change issue involvement and preferred societal response by segment and overall: 2011 and 2016.

	Year	Alarmed	Concerned	Cautious	Disengaged	Doubtful	Dismissive	Overall	Scale Range
Rating of whether climate change is a good thing or a bad thing^a^	2011 2016 *% Change*	5.71 5.75 *0*.*9%*	4.95 5.06 *2*.*0%*	4.33 4.59 *5*.*9%*	3.70 3.49 *-5*.*6%*	3.66 3.71 *0*.*3%*	3.29 3.23 -*2*.*1%*	4.45 4.61 *3*.*5%*	6
How worried you are about climate change^b^	2011 2016 *% Change*	3.62 3.64 *0*.*6%*	3.02 3.11 *2*.*9%*	2.48 2.52 *1*.*41%*	2.47 2.81 ***13*.*6%***	1.89 1.85 *-2*.*0%*	1.30 1.24 *-4*.*1%*	2.59 2.75 *6*.*4%*	4
How much you have thought about climate change before today^c^	2011 2016 *% Change*	3.70 3.68 *-0*.*6%*	2.91 2.81 *-3*.*3%*	2.28 2.19 *-3*.*9%*	2.70 2.42 ***-10*.*2%***	2.58 2.27 ***-11*.*9%***	2.74 2.38 ***-13*.*3%***	2.82 2.69 *-4*.*8%*	4
Need more information before making up mind about climate change^d^	2011 2016 *% Change*	2.86 2.72 *-4*.*9%*	2.30 2.30 *0*.*0%*	2.03 2.08 *2*.*4%*	2.30 2.37 *3*.*1%*	2.59 2.51 *-3*.*0%*	3.33 3.47 *4*.*0%*	2.49 2.44 *-2*.*3%*	4
Importance to you personally^e^	2011 2016 *% Change*	4.44 4.36 *-1*.*8%*	3.35 3.39 *1*.*3%*	2.63 2.59 *-1*.*3%*	2.91 3.01 *3*.*5%*	2.14 2.00 *-6*.*6%*	1.68 1.51 ***-9*.*9%***^*#*^	2.96 3.04 *2*.*8%*	5
Could easily change your mind about climate change^f^	2011 2016 *% Change*	1.29 1.34 *3*.*9%*	2.06 2.08 *0*.*9%*	2.64 2.54 *-3*.*5%*	2.64 2.71 *3*.*0%*	2.33 2.46 *5*.*7%*	1.72 1.71 *-0*.*5%*	2.11 2.15 *1*.*8%*	5
Personally experienced effects of climate change^f^	2011 2016 *% Change*	3.07 3.12 *1*.*6%*	2.53 2.66 *5*.*4%*	2.07 2.21 *6*.*9%*	2.39 2.75 ***14*.*7%***	1.76 1.92 ***9*.*4%***	1.36 1.38 *1*.*4%*	2.26 2.48 ***9*.*6%***	4
How often discuss with family and friends^g^	2011 2016 *% Change*	3.15 3.14 *-0*.*4%*	2.60 2.53 *-2*.*9%*	1.94 1.74 ***-10*.*3%***	2.64 2.58 *-2*.*3%*	2.22 1.84 ***-17*.*5%***	2.35 1.80 ***-23*.*5%***	2.48 2.33 *-5*.*8%*	4
How many of your friends share your views on climate change^h^	2011 2016 *% Change*	3.53 3.65 *3*.*4%*	2.91 3.02 *3*.*9%*	2.23 2.16 *-3*.*1%*	2.95 2.85 *-3*.*4%*	3.01 2.41 ***-19*.*9%***	3.44 3.25 *-5*.*4%*	2.95 2.86 *-3*.*2%*	5
Priority for Prime Minister and Parliament^i^	2011 2016 *% Change*	3.79 3.77 *-0*.*5%*	3.07 3.07 *0*.*0%*	2.37 2.28 *-4*.*0%*	2.16 2.29 *6*.*0%*	1.51 1.44 *-4*.*2%*	1.03 1.01 *-2*.*0%*	2.49 2.59 *3*.*9%*	4
Corporations or industry should be doing more or less to address climate change^j^	2011 2016 *% Change*	4.86 4.88 *0*.*5%*	4.37 4.31 *-1*.*4%*	3.99 3.88 *-2*.*7%*	3.66 3.00 ***-18*.*2%***	3.37 3.08 *-8*.*4%*	2.60 2.51 -*3*.*5%*	3.95 3.89 *-1*.*5%*	5
Citizens themselves should be doing more or less to address climate change^j^	2011 2016 *% Change*	4.76 4.73 *-0*.*6%*	4.23 4.17 *-1*.*6%*	3.85 3.78 *-1*.*8%*	3.45 3.07 ***-11*.*2%***	3.21 2.97 *-7*.*2%*	2.53 2.43 *-4*.*3%*	3.82 3.79 *-0*.*8%*	5
Effort Australia should make to reduce global warming given associated costs^k^	2011 2016 *% Change*	3.72 3.66 *-1*.*5%*	3.20 3.17 *-1*.*2%*	2.88 2.82 *-2*.*1%*	2.58 2.63 *2*.*1%*	1.98 2.00 *1*.*1%*	1.30 1.28 *-1*.*0%*	2.76 2.85 *3*.*1%*	4
Australia should reduce its emissions regardless of what others do (% agree)	2011 2016 *% Change*	98% 96% *-1*.*3%*	89% 85% *-3*.*9%*	74% 77% *3*.*3%*	40% 20% ***-50*.*3%***	26% 39% ***51*.*3%***	12% 12% *-1*.*9%*	66% 69% *4*.*3%*	—

^a^ 1—very good, …, 6 –very bad

^b^ 1 –not at all worried, 2-not very worried, 3 somewhat worried, 4 –very worried

^c^ 1 –not at all, …, 4 –a lot

^d^ 1 –I need a lot more information, …, 4 –I do not need any more information

^e^ 1 –not at all important, 5 –extremely important

^f^ 1 –strongly disagree, 4 –strongly agree

^g^ 1 –never, …, 4 –very often

^h^ 1 –none, …, 5 –all

^i^ 1 –low, 2 –medium, 3 –high, 4 –very high

^j^ 1 –much less, 2 –less, 3 –currently doing the right amount, 4 –more, 5 –much more

^k^ 1 –no effort, 2 –a small-scale effort even if it has small economic consequences, 3 –a medium-scale effort even if it has moderate economic consequences, 4 –a large-scale effort even if it has large economic consequences.

# nonsignificant difference, all other shaded cells have significant differences at p<0.05 or higher

**Table 4:** Climate change energy use behaviours by segment and overall: 2011 and 2016.

	Year	Alarmed	Concerned	Cautious	Disengaged	Doubtful	Dismissive	Overall	Scale Range
Over past 12 months how often have contacted Government Officials to urge them to take action to reduce global warming^a^	2011 2016 *% Change*	1.93 1.86 *-3*.*5%*	1.29 1.26 *-2*.*1%*	1.00 1.00 *0*.*0%*	1.61 1.92 ***19*.*0%***	1.02 1.01 *-1*.*1%*	1.04 1.01 *-2*.*9%*	1.29 1.33 *3*.*3%*	5
Over past 12 months how often have rewarded companies taking steps to reduce global warming by buying their products^a^	2011 2016 *% Change*	3.48 3.35 *-3*.*5%*	2.64 2.34 ***-11*.*1%***	1.32 1.17 *-11*.0%^*^	2.64 2.36 ***-10*.*7%***	1.31 1.16 ***-10*.*9%***^***#***^	1.07 1.00 *-6*.*8%*	2.14 2.04 *-4*.*3%*	5
Over past 12 months how often have punished companies opposing steps to reduce global warming by not buying their products^a^	2011 2016 *% Change*	3.51 3.39 *-3*.*6%*	2.37 2.11 ***-11*.*3%***	1.08 1.05 *-3*.*0%*	2.41 2.13 ***-11*.*6%***	1.03 1.07 *3*.*9%*	1.03 1.00 *-2*.*5%*	1.92 1.89 *-1*.*5%*	3
Intention over next 12 months to buy products of companies taking steps to reduce global warming^b^	2011 2016 *% Change*	2.82 2.78 *-1*.*2%*	2.59 2.47 *-4*.*7%*	2.09 2.04 *-2*.*0%*	2.25 2.08 *-7*.*7%*	2.01 1.95 *-3*.*3%*	1.91 1.88 *-1*.*8%*	2.33 2.28 *-2*.*2%*	5
Intention over next 12 months to punish companies opposing steps to reduce global warming by NOT buying their products^b^	2011 2016 *% Change*	2.85 2.76 *-3*.*1%*	2.50 2.37 *-5*.*2%*	2.07 2.02 *2*.*5%*	2.19 1.98 ***-9*.*5%***	1.98 1.94 *-1*.*8%*	1.89 1.84 *-2*.*7%*	2.29 2.23 *-2*.*8%*	3
How often reduce thermostat in winter and plans to increase or decrease this over next 12 months^c^	2011 2016 *% Change*	6.42 5.14 ***-19*.*9%***	5.58 4.47 ***-20*.*0%***	4.58 3.26 ***-28*.*9%***	5.61 5.21 *-7*.*1%*	4.68 3.25 ***-30*.*6%***	3.71 3.01 ***-18*.*9%***	5.14 4.18 ***-18*.*8%***	10
How often use public transportation or car pool and plans to increase or decrease this over next 12 months^c^	2011 2016 *% Change*	5.83 6.16 *5*.*7%*	4.93 5.27 *7*.*0%*	3.69 4.21 ***14*.*0%***	5.05 5.47 *8*.*2%*	3.84 3.91 *1*.*9%*	3.48 2.97 *-**14*.5%^*^	4.53 4.92 *8*.*8%*	10
How often walk or use bike instead of driving and plans to increase or decrease this over next 12 months^c^	2011 2016 *% Change*	6.14 5.89 *-3*.*9%*	5.24 5.30 *1*.*1%*	4.09 4.00 *-2*.*3%*	5.29 5.44 *2*.*7%*	4.06 4.00 *-1*.*4%*	3.80 3.80 *0*.*0%*	4.83 4.88 *1*.*1%*	10

^a^ 1 –never, 2 –once, 3 –a few times (2–3), 4 –several times (4–5), 5 –many times (6+)

^b^ 1 –less frequently, 2 –about the same, 3 –more frequently

^c^ 1 –never do this and intend to do the same, 2 –never do this and intend to do more often, …, 10 –always do this and intend to do more often.

* significant difference at p<0.10

# nonsignificant difference, all other shaded cells have significant differences at p<0.05 or higher

### Changes in segment size

**Fig 1:**
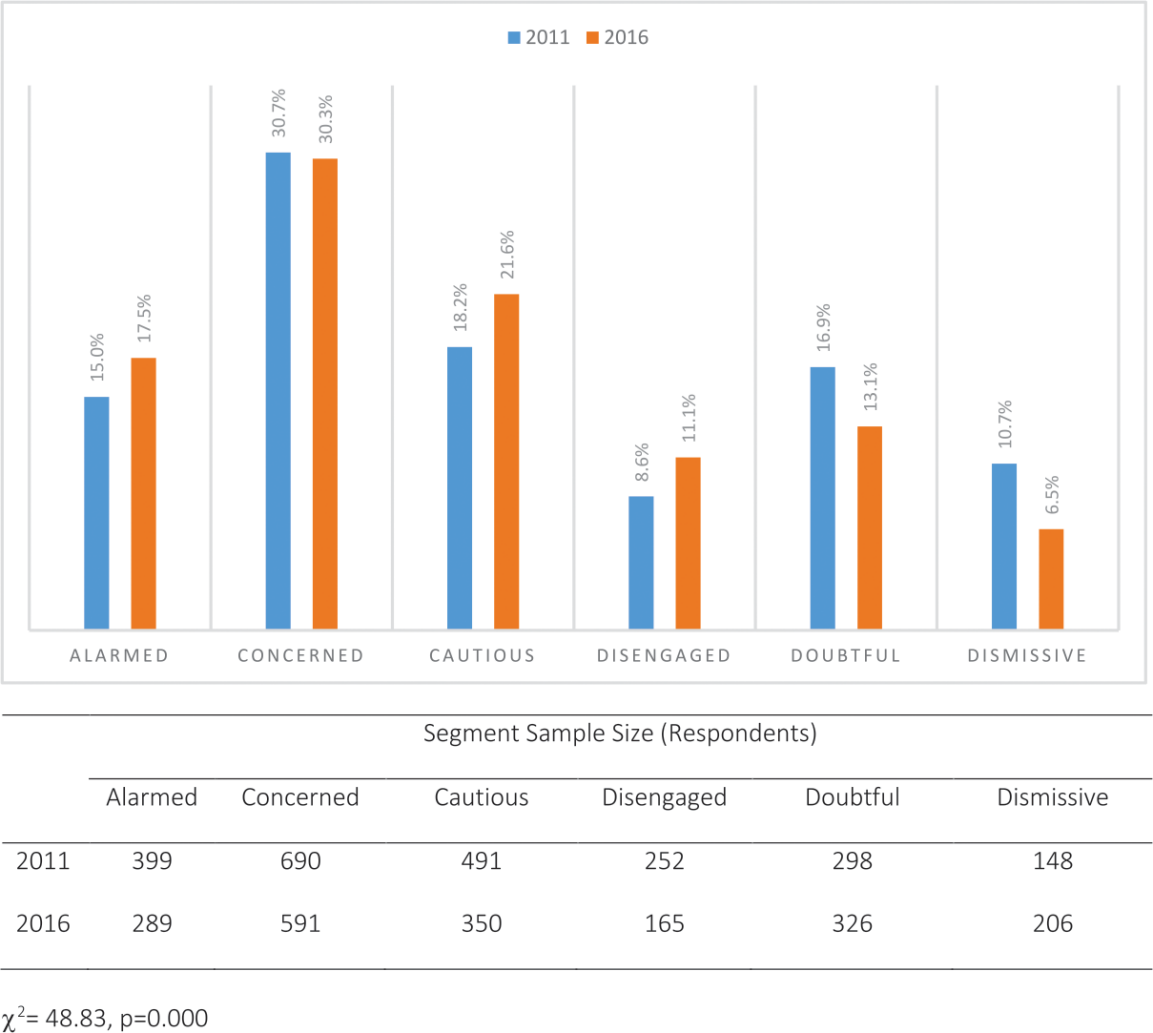
Segment size: 2011 vs 2016.

Fig 1 shows the proportion of respondents falling into the six different segments in 2011 and 2016. The key result is that there has been a leftward movement during the period towards the *Alarmed* and *Concerned* categories and away from the *Doubtful* and *Dismissive* groups. With a Chi-square value of 48.83, the difference is statistically significant. While there is an increased proportion in the *Alarmed* group (from 15.0% to 17.5%), the major shifts are out of the *Doubtful* and *Dismissive* groups and into the *Cautious* and *Disengaged* groups. With 32.7% now in these latter two groups (up from 26.8%), there would be a higher likelihood of climate change messages being effective because Morrison et al. [16] showed that members of these two groups are likely to have their opinions on climate change issues easily changed.

### Beliefs about climate change

The results in Table 2 for both 2011 and 2016 on beliefs about climate change in Australia confirm that the categories can be ranked from *Alarmed* to *Dismissive*. The responses for both years in general have a response profile that is increasing or decreasing across the six segments. For example, the *Alarmed* and *Concerned* segments have higher proportions certain that climate change is occurring and has human causation, whereas the *Doubtful* and *Dismissive* segments have low proportions with these views. The *Alarmed* tend to view themselves as being harmed by climate change, and have a more positive view of the efficacy of individual and collective action to combat the problem. To assist in reading the results in Table 2 (and Tables 3 to 5 as well), where there have been percentage changes in the variables of at least 10% between 2011 and 2016, the cells have been shaded grey.

**Table 5:** Socio-demographic profile of the Australian segments.

		Alarmed	Concerned	Cautious	Disengaged	Doubtful	Dismissive
Age (years)	2011 2016	45.6 47.9	45.5 44.8	44. 7 46.6	47.8 39.5	51.3 52.5	53.2 56.4
Gender (% Male)	2011 2016	43% 40%	41% 44%	43% 42%	57% 58%	57% 61%	72% 61%
Degree qualified	2011 2016	50% 50%	37% 45%	32% 34%	41% 52%	32% 31%	30% 36%
Trade qualified	2011 2016	18% 24%	26% 22%	29% 26%	27% 16%	29% 27%	33% 30%
Year 12 or below qualification	2011 2016	20% 20%	24% 24%	36% 33%	24% 25%	31% 37%	31% 31%
Income	2011 2016	$75,499 $76,991	$72,417 $75,187	$75,592 $73,692	$71,940 $84,307	$71,781 $75,169	$82,326 $77,331
**Location:** Capital City	2011 2016	61% 66%	59% 64%	57% 64%	58% 72%	53% 55%	52% 61%
Regional Town	2011 2016	26% 21%	26% 23%	30% 26%	26% 20%	35% 33%	33% 24%
Rural Area	2011 2016	13% 13%	12% 12%	13% 10%	15% 8%	11% 12%	15% 14%
**Employment status:** Employed full-time	2011 2016	38% 30%	32% 30%	39% 32%	36% 48%	33% 30%	38% 33%
Employed part-time or casual	2011 2016	18% 18%	17% 20%	15% 16%	9% 19%	16% 16%	12% 10%
Self employed	2011 2016	4% 7%	5% 4%	7% 4%	7% 6%	7% 8%	10% 9%
Unemployed—looking for work	2011 2016	5% 4%	5% 5%	5% 6%	5% 4%	3% 3%	1% 3%
Student full-time or part-time	2011 2016	5% 9%	6% 9%	3% 7%	3% 6%	1% 2%	1% 1%
Home duties / not in paid employment—not looking for work	2011 2016	10% 8%	13% 10%	13% 12%	15% 7%	8% 8%	7% 10%
Retired / pension recipient	2011 2016	20% 25%	20% 21%	19% 24%	24% 12%	30% 33%	29% 24%
**Voting Behaviour:** Labor	2011 2016	37% 33%	37% 31%	25% 27%	20% 22%	10% 16%	9% 9%
Liberal	2011 2016	9% 13%	17% 24%	32% 28%	32% 27%	54% 40%	62% 51%
National	2011 2016	1% 1%	1% 3%	2% 2%	3% 8%	8% 6%	5% 7%
Greens	2011 2016	36% 28%	14% 11%	4% 3%	5% 7%	0% 2%	
I have no interest in politics	2011 2016	13% 13%	19% 19%	25% 25%	22% 14%	19% 17%	18% 13%
I'd prefer not to say	2011 2016	5% 7%	11% 10%	12% 11%	15% 20%	9% 13%	6% 12%

For one item in Table 2 –the belief that new technology will solve the climate change problem–the *Disengaged* segment has the highest score (again for both 2011 and 2016). Also this group has unusually high scores for the belief that their own actions will reduce their personal contribution to climate change.

Turning to changes that have occurred from 2011 to 2016, the results of Table 2 indicate that, on average, a higher proportion believes that (a) climate change is happening (though only for the *Dismissive* group has there been a significant change); (b) it is caused by human activities (significant change across all groups except the *Dismissive* group); and (c) there is consensus among scientists (significant change across all groups except the *Alarmed* group). Of the ten remaining beliefs shown in Table 2 there was a statistically significant change for either only one segment (four cases) or no change (six cases). Also, the category that had the greatest number of statistically significant changes (five) was the *Dismissive* group. While all five of these changes were towards engagement with climate change, they were from a low base so that this group still effectively remained detached in 2016.

An overall summary of the results of Table 2 is that belief in climate change has increased marginally since 2011, but more substantially for increased belief in human causation and agreement between scientists.

### Personal involvement in the issue

There are five questions in Table 3 about personal involvement in climate change issues that do not follow the monotonically increasing or decreasing pattern generally observed in Table 2. The first three of these show that those who desire more information, have friends who share their views and are unwilling to change their opinions are at either end of the spectrum in the *Alarmed* and *Dismissive* categories. This confirms the observation made in Morrison et al [16] that those in the categories *Cautious* and *Disengaged* are more open to change and hence to being influenced by a targeted marketing message. The other two questions that do not portray monotonic change are on how much respondents have thought about climate change and how often they discuss the issue with family and friends. There is no obvious pattern across the categories for these two questions.

Turning to the issue of change between 2011 and 2016, the overall impression is one of reduced involvement, and in general a preference for a less aggressive response. All groups had a reduction in how much they had thought about climate change before today and how often they discuss the issue with family and friends, with these changes being statistically significant for three of the six categories. This is despite an almost 10% increase in respondents personally experiencing the effects of climate change.

In terms of action, all groups considered that corporations and citizens should be doing less to address climate change in 2016 than in 2011, though only for the *Disengaged* group were these changes statistically significant. Indeed, this group has the highest number of significant changes for the issues covered in Table 3. These changes show that the *Disengaged* group are more worried in 2016 about climate change and are more likely to have personally experienced the effects of climate change. Nevertheless, the other statistically significant changes show that a smaller proportion of this group in 2016 also considers that corporations and citizens should do more to address climate change, and a similar smaller proportion considers that Australia should reduce its emissions regardless of what others do. The *Disengaged* group also has a statistically significant reduction between 2011 and 2016 in how much they have thought about climate change issues.

The decline in belief that corporations or industry or citizens should be doing more about climate change, or that Australia should unilaterally reduce its emissions, among members of the *Disengaged* segment most likely also reflects changes in the membership of the segment rather than just changes in the views of those who were *Disengaged* members in 2011. Results presented in Fig 1 indicated that membership of the *Cautious* and *Disengaged* segments increased while membership of the *Doubtful* and *Dismissive* segments declined. Thus some respondents that were in 2011 would have been classified as *Doubtful* or *Dismissive* have shifted in 2016 to become either *Cautious* or *Disengaged*. It is probable that this is because they have now begun to experience the effects of climate change. However, these respondents may still have the attitude generally held by those in the *Doubtful* and *Dismissive* segments that individuals and business are not responsible to act to reduce climate change and that the government should not act unilaterally. If so, this would reduce the means for these variables and lead to this result. The other possible explanation is that others in this segment have changed their view over time regarding who should be responsible and the actions that Australia should take; however, we regard the former as the more plausible explanation.

An overall summary of the results is that there is more concern about climate change, particularly as reflected in the movement of households into groups expressing higher concern (Fig 1), even though there is little change in the mean level of concern within each group (Table 3). In other words, the general trend is for households to increase their level of concern and for some to consequently move to groups portraying more concern on average. However, those who do so move would be expected to enter their new group with scores lower than mean for this group, and thereby suppress any tendency for the group mean to rise. There is also more experiencing of climate change despite the fact that a larger proportion of respondents have thought less about the issue. Also there is a higher reluctance to address climate change.

### Energy use behaviours

Every question in Table 4 (on energy use behaviours in both 2011 and 2016) follows a declining trend when you move from the *Alarmed* through to the *Dismissive* category. Hence, the *Alarmed* are more likely to reward companies that reduce emissions and punish companies that do not. They are also more interested in carpooling, bicycling and adjusting thermostats. In two areas–thermostat use and intention to reward companies that reduce emissions–there has been a decline between 2011 and 2016 for all groups, with a statistically significant decline for five of the six groups on thermostat use, and for three of the six groups in relation to rewarding companies that reduce emissions. While from an Australian perspective these two seem like US-centric activities, especially related to heating where, for many years, the US media has emphasised regulation of thermostats, the decline since 2011 still indicates an area of concern in Australia. It is possible that this decline is a rebound effect related to the increased use of renewable energy [30]; given that Australians have substantially increased their reliance on rooftop solar panels during the past five years (from about 308,000 households with panels in January 2011 to almost 1.5 million in January 2017) [31], they may be inclined to use more electricity to maintain household comfort. Another possibility is that the result is due to 2011 survey occurring during winter, while the 2016 survey occurred during summer, and this differing context may have affected results.

The response to a number of other questions in Table 4 shows a lower emphasis on behaviours to reduce climate change impacts. All four questions on actions in relation to companies taking steps to (or opposing steps to) reduce their climate change impact show a reduced commitment to take action between 2011 and 2016, though not all changes are statistically significant. The intention to walk or ride a bike instead of driving shows little change from 2011 and 2016. About the only area that shows a minor positive change in energy use behaviour is the intention to take public transport or car pool.

### Sociodemographic profile of Australian segments

The *Alarmed* end of the spectrum had a higher proportion of degree-qualified individuals in both 2011 and 2016, while the *Dismissive* end had a higher proportion of trade qualified (Table 5). However, the educational distinctions between the groups were not so clear in 2016. The *Concerned* group has had a statistically significant increase in degree qualified individuals and the *Disengaged* group has had a significant increase in both degree and trade qualified individuals.

In both 2011 and 2016 the *Dismissive* end tended to be older, male, live in less urbanised areas and are more likely to be self-employed. When classified by political party preference, the Greens and Labor (both progressive parties) have a higher proportion of *Alarmed* and *Concerned*, while those expressing a preference for Liberal or National (both conservative parties) are more likely to be *Doubtful* and *Dismissive*, consistent with findings in the US [32, 33, 34] and Australia [35]. Nevertheless there has been a distinct movement so that a higher proportion of the *Alarmed* and *Concerned* were Liberal voters in 2016 than in 2011 and a lower proportion of the *Doubtful* and *Dismissive* were Liberal voters. These two sets of changes over time are statistically significant. This result points to a convergence of views on climate change issues of supporters of the major political parties.

The characteristics of the middle segments, *Cautious* and *Disengaged*, are likely to be of particular interest to policy-makers, given their willingness to change their opinion about climate change [21]. The changes in the characteristics of the *Disengaged* segment from 2011 to 2016 were substantial. While they tended to be slightly older than the overall sample mean in 2011, they became the youngest segment in 2016. They recorded significant changes in degree qualified (increase), trade qualified (decrease), income (increase from lowest to highest category), rural to urban, being employed full or part-time or casual (increase), and voting intentions towards the National party (increase), though they still remained by far the segment having the highest proportion of people who indicate no interest in politics or would not indicate their voting intentions.

## Discussion

The challenge of engaging households in climate change issues has led to the widespread adoption of social marketing techniques such as the use of segmentation to better understand the different household types within the community, and how to more effectively tailor the case for climate change action for each [2, 1]. However, segments are not static, and they change in their size and their characteristics. For this reason, the Yale/George Mason Group have investigated changes in the Six Americas segments over time, and in Australia Leviston et al. [36] examined changes in a range of individual climate change attitudes and behaviours over a five-year period (2010–2014), though they did not examine changes in household segments.

Our results indicate that most Australian households now consider that climate change is occurring, and that almost two-thirds consider that it is mostly caused by humans. Further, compared to 2011, in our most recent 2016 survey, households overall are more likely to believe that they have experienced the effects of climate change and they think the effects are more imminent. They are also more likely to believe that climate change is human induced and that there is consensus between scientists. However, the former view is only held by at least 50% of households in 3 out of 6 segments, while the latter view is only held by at least 50% of households in 2 out of 6 segments. The increasing belief in human induced climate change is consistent with Leviston et al. [34] who reported minor increases between 2011 and 2014 in belief in human induced climate change, though they also reported a drop between 2010 and 2011 in this belief.

We also see a trend in segment membership away from the *Doubtful* and *Dismissive* segments (down from 27.8% to 19.6%) of households and an increase in the proportion of respondents who are in the *Cautious* and *Disengaged* segments (up from 26.8% to 32.7%), though the proportion of households in the *Alarmed* and *Concerned* segments is relatively stable. Together these findings are suggestive that community awareness of climate change and acceptance of the basic facts are both increasing.

Although more Australians report experiencing effects of climate change and are expressing greater concern about the issue, issue fatigue appears to be increasing, particularly among households not in the *Alarmed* and *Concerned* segments. This can be seen in the reduced issue involvement of multiple segments, with reductions in how much they had thought about climate change before today and how often they discuss the issue with family and friends. It is also seen in a decline in household willingness to address climate change. Apart from increased use of public transport, household efforts to mitigate emissions are static or declining, and households overall do not believe that they should be doing more. This applies especially to the members of the *Cautious* and *Disengaged* segments. Similarly, there is a decreasing will to reward or punish companies for their climate change behaviours. Regarding what the government should do, households are on average converging on the view that Australia should make a “medium scale effort even if it has moderate economic consequences”. Thus, overall, households increasingly believe in climate change, are experiencing it and are more worried about it, but they do not think that they or businesses should do much more than they already are about it, and that Australia should only make a moderate effort to deal with it. The country appears to be at risk of entering into a state of resigned acceptance.

This emerging evidence of issue fatigue suggests that efforts are needed to keep households going in their efforts to reduce climate emissions and adapt to climate change. Messages could seek to reinforce personal efficacy, and to provide positive reasons such as health, social welfare, economic and other community benefits for maintaining efforts to reduce carbon emissions [23, 3]. Innovative and creative appeals that draw on the emotions may be more effective than cognitively based appeals [22, 37], or use of strategies that entertain while seeking to change behaviour [25]. Recent internet-based experiments reported by van der Linden et al. [38] which were designed to inoculate sub-populations of concern against misinformation campaigns used both affective and cognitive messaging. These experiments sought to inform participants about the degree of scientific consensus and the nature of misinformation campaigns, and were found to be effective at neutralising the harmful effects of misinformation. Public scorecards could also be used to remind households (and businesses) about progress with respect to energy use and use of public transport, similar to currently-used reminders about domestic water usage and reservoir water levels. Both members of *Cautious* and *Disengaged* segments indicate a willingness to change their minds about climate change, so despite their views that they do not need to change their current level of effort, information campaigns about what they should be doing and why are increasingly likely to resonate given their growing experience of and belief in climate change.

In summary, there are both positive and negative findings from the two surveys. Positively, there is an increased acceptance of climate change and its importance, and higher concern, shown particularly by movements in membership of segments from the *Dismissive* end toward the *Alarmed* end (though not in changes over time in the average scores in each segment). However, there is also evidence of decreased issue involvement. This implies a need to move beyond using strategies to increase awareness and acceptance of climate change, to developing and using strategies to keep people focused on efforts to address climate change.
